# Medical Tourism and Telemedicine: A New Frontier of an Old Business

**DOI:** 10.2196/jmir.5432

**Published:** 2016-05-23

**Authors:** Yan Alicia Hong

**Affiliations:** ^1^ School of Public Health Xiamen University Xiamen China; ^2^ School of Public Health Texas A&M University College Station, TX United States

**Keywords:** E-hospital, medical tourism, telemedicine, ethics

## Abstract

In October 2015, the “Chinese American Physicians E-Hospital” celebrated its “grand opening” online. All physicians affiliated with this E-Hospital are bilingual Chinese American physicians, who provide services ranging from initial teleconsulting to international transfer and treatment in the United States. Such telemedicine platform for medical tourism not only saves the patients from the hassles of identifying and connecting with an appropriate health service provider but also minimizes the language and cultural barriers. As a growing number of patients from middle- and low-income countries travel to the United States (US) for medical care, we face promising opportunities as well as mounting challenges. The Centers for Disease Control (CDC) in the US has guidance for Americans seeking care overseas, but is not available for international patients seeking care in US. This article opens a dialogue on the challenges associated with flourishing medical tourism and telemedicine, including quality assessment, risk communication, ethical guidelines, and legal concerns.

In October 2015, the “Chinese American Physicians E-Hospital” celebrated its “grand opening.” Designed specifically for the Chinese population, this E-Hospital launched a website only in Chinese and offers toll free numbers for Chinese patients in both North America and China; it also provides online customer service for Chinese people around the world. All physicians affiliated with this E-Hospital are bilingual Chinese American physicians, who are board certified in the US. These highly qualified and culturally fit physicians provide initial teleconsulting, joint consultation, and ultimately, international transfer. This telemedicine platform for medical tourism not only saves the patients from the hassles of identifying and connecting with an appropriate health services provider but also eliminates the language and cultural barriers.

Medical tourism is defined as traveling to a foreign country to seek medical care. A 2013 online survey from US reported that 27% of patients had engaged in some form of medical tourism. The number of traveling patients and cost for medical tourism has skyrocketed in the recent years, contributing to a flourishing business of $439 billion [1]. Traditionally, medical tourists travel from high-income countries to middle- and low-income countries to seek comparable or identical care at a lower price. Countries such as China, India, Mexico, and Thailand have long been prominent destinations for Western medical tourists [2].

With a growing wealthy class in the middle-income countries and rapid penetration of the Internet, the “one-way flow” of medical tourists has been gradually replaced with “two-way exchange.” More and more patients from middle- and low-income countries travel to the high-income countries for better diagnostic capabilities, state-of-the-art medical technologies, and advanced treatment options that may not be available in their home countries. The prominent hospitals like the Mayo clinic, M.D. Anderson Cancer Center, and Cleveland Clinic are among the most sought-after clinics and hospitals in the US.

A recent report revealed that the number of Chinese patients seeking medical care in US has increased by 400% from 2004 to 2014, spurring a billion dollar business [3]. A new WHO report showed that China’s cancer incidence was in rise and accounted for 21.8% of the global total of cancer diagnoses and 26.9% of world’s total cancer deaths [4]. Currently, the 5-year cancer survivor rate in China is about 30%, compared to 66% in US. [5]; most clinical trials and new treatments are only available in a selected number of renowned hospitals in US. More than 70% of these Chinese patients seek oncological treatments in US; they typically spend $100,000-$150,000 for their medical trip and pay for their treatment with cash upfront [3]. The aforementioned are the driving forces behind the spurring medical tourism in US [6].

In the wake of high profit in medical tourism, many hospitals are investing to make their services more visible and convenient to their clients from overseas; many hospitals have a department dedicated to provide services to these international patients [3,6]. As the US hospitals and health systems invest in population health initiatives and commit to new payment models that align incentives for keeping patients away from the hospital and clinics, these medical tourists seems to be a logical strategy for keeping hospital beds filled with a new type of fee-for-service customers. E-hospital certainly serves as an efficient platform to attract more medical tourists, and we expect to see more international patients in American hospitals. Accompanying the promising opportunities are the mounting challenges associated with growing number of medical tourists in US.

The first challenge is a lack of reliable assessment of healthcare quality across borders, including a scale of price to outcome. Increasing complexity of healthcare provision and skyrocketing cost of medical care requires more transparency in pricing and standardization of quality assessment. For international patients, a reliable assessment system comparable across borders is especially needed. As Shaw advocated, we need to standardize healthcare standards, and efforts are required in statutory regulation, institutional licensing and accreditation, and increasing transparency of healthcare pricing [7].

Second, there is insufficient communication on the risks associated with medical tourism. The CDC has guidance for Americans seeking care overseas [8], but similar guidance is not available to international patients seeking care in US. Analysis of 91 medical tourism brokers’ websites in North America found that most websites failed to report any procedural, postoperative, or legal concerns associated with their services [9]. Analysis of media discourses on medical tourism indicated that risks were less communicated than benefits [10]. Regulations on these medical tourism broker services including E-Hospitals are lagging behind the rapid development of telemedicine and are urgently needed.

Third, there are inadequate policies on ethical concerns related to clinical trials. International patients, traveling thousands of miles to seek treatment in US, are often charged with full emotion and high hope; many are recruited into clinical trials for new but risky treatments [10]. How to address the language and cultural barriers for these patients and how to ensure they understand the potential risks associated with clinical trials have not been addressed in current rules and policies.

Fourth, growing legal concerns are associated with medical tourists. Parallel to the growing number of medical tourists are the more frequent reports of lawsuits associated with medial tourism. Police aids of “maternity hotels” targeting Chinese women giving birth in U.S. and big-dollar lawsuits associated with malpractice in treating international patients have often made headlines. Some of these incidents were attributed to limited communication of risks and survivor rates. Many “gray zones” in medical tourism have not been addressed in our current legislature but need immediate attention.

Rapid growth of medical tourism mirrors accelerative globalization. As Crisp vividly describes “turning the world upside down,” ubiquitous Internet access has enabled patients around the world to seek the best care available and facilitates efficient communication of medicine globally [11]. But how to ensure the patient safety, quality of care, ethical issues, and legal concerns remain inadequately addressed, for patients, healthcare providers, and policy makers across borders. Data are the very foundation for the making evidence-based policies and practices; but in our review of literature on medical tourism, we found a dearth of such data. As we enter a new frontier of telemedicine for the old business of medical tourism, we need more research and dialogue on the issues and impact associated with the evolving models of medical consumerism.

## References

[1] American Medical Tourism Association.

[2] Hall CM (2012). Medical Tourism: The Ethics, Regulation, and Marketing of Health Mobility. Routledge.

[3] The Wall Street Journal.

[4] (2014). World Health Organization.

[5] Zeng H, Zheng R, Guo Y, Zhang S, Zou X, Wang N, Zhang L, Tang J, Chen J, Wei K, Huang S, Wang J, Yu L, Zhao D, Song G, Chen J, Shen Y, Yang X, Gu X, Jin F, Li Q, Li Y, Ge H, Zhu F, Dong J, Guo G, Wu M, Du L, Sun X, He Y, Coleman MP, Baade P, Chen W, Yu XQ (2015). Cancer survival in China, 2003-2005: a population-based study. Int J Cancer.

[6] Chang J Chinese spur medical-tourism growth in the US.

[7] Shaw, CD (2015). How can healthcare standards be standardised?. BMJ Quality & Safety.

[8] Gaines J, Nyugen D (2014). CDC Yellow Book information on Medical Tourism.

[9] Jun J, Oh KM (2015). Framing risks and benefits of medical tourism: a content analysis of medical tourism coverage in Korean American community newspapers. J Health Commun.

[10] Henthorne Tl, George (2009). The Incorporation of Telemedicine With Medical Tourism: A Study of Consequences. Journal of Hospitality Marketing & Management.

[11] Crisp N (2010). Turning the World Upside Down: The Search for Global Health in the 21st Century.

